# An Indigenous-informed scoping review study methodology: advancing the science of scoping reviews

**DOI:** 10.1186/s13643-024-02586-1

**Published:** 2024-07-15

**Authors:** Wanda Phillips-Beck, Bryden L. J. Bukich, Kellie Thiessen, Josée G. Lavoie, Annette Schultz, Julianne Sanguins, Geraldine Beck, Brenda Longclaws, Geraldine Shingoose, Matta Palmer, Janice Linton, Bekelu Negash, Taylor Morriseau

**Affiliations:** 1 First Nations Health and Social Secretariat of Manitoba (FNHSSM), Winnipeg, Canada; 2 College of Nursing, Rady Faculty of Health Sciences, University of Manitoba, Winnipeg, Canada; 3 Department of Community Health Sciences, Rady Faculty of Health Sciences, University of Manitoba, Winnipeg, Canada; 4 Manitoba Metis Federation, Winnipeg, Canada; 5 Rady Faculty of Health Sciences, Neil John Maclean Health Sciences Library, University of Manitoba, Winnipeg, Canada

**Keywords:** Indigenous scoping review methodology, Indigenous research methodology

## Abstract

**Background:**

Historically, Indigenous voices have been silent in health research, reflective of colonial academic institutions that privilege Western ways of knowing. However, Indigenous methodologies and methods with an emphasis on the active involvement of Indigenous peoples and centering Indigenous voices are gaining traction in health education and research. In this paper, we map each phase of our scoping review process and weave Indigenous research methodologies into Arksey and O’Malley’s (2005) framework for conducting scoping reviews.

**Methods:**

Guided by an advisory circle consisting of Indigenous Knowledge Keepers and allied scholars, we utilized both Indigenous and Western methods to conduct a scoping review. As such, a circle of Knowledge Keepers provided guidance and informed our work, while our methods of searching and scoping the literature remained consistent with PRISMA-ScR guidelines. In keeping with an Indigenous methodology, the scoping review protocol was not registered allowing for an organic development of the research process.

**Results:**

We built upon Arksey and O’Malley’s 5-stages and added an additional 3 steps for a combined 8-stage model to guide our research: (1) Exploration and Listening, (2) Doing the Groundwork, (3) Identifying and Refining the Research Question, (4) Identifying Relevant Studies, (5) Study Selection, (6) Mapping Data, (7) Collating, Summarizing and Synthesizing the Data, and lastly, (8) Sharing and Making Meaning. Engagement and listening, corresponding to Arksey and O’Malley (2005)’s optional “consultation stage,” was embedded throughout, but with greater intensity in stages 1 and 8.

**Conclusion:**

An Indigenous approach to conducting a scoping review includes forming a team with a wide array of experience in both Indigenous and Western methodologies, meaningful Indigenous representation, and inclusion of Indigenous perspectives to shape the analysis and presentation of findings. Engaging Indigenous peoples throughout the entire research process, listening, and including Indigenous voices and perspectives is vital in reconciliation research, producing both credible and useable information for both Indigenous communities and academia. Our Indigenous methodology for conducting a scoping review can serve as a valuable framework for summarizing Indigenous health-related research.

**Supplementary Information:**

The online version contains supplementary material available at 10.1186/s13643-024-02586-1.

## Background

To redress the legacy of residential schools and advance the process of Canadian reconciliation, the federal government officially established the Truth and Reconciliation Commission (TRC) of Canada in June of 2008 to document the history and lasting impacts of the Canadian Indian residential school system on Indigenous students and their families. The TRC recognized the pernicious relationship between racism and educating our healthcare professionals [1]. As such, #24 of the 94 Calls to Action specifically called on “medical and nursing schools in Canada to require all students to take a course dealing with Aboriginal health issues, including the history and legacy of residential schools, the United Nations Declaration on the Rights of Indigenous Peoples, Treaties and Aboriginal rights, and Indigenous teachings and practices. This also included skills-based training in intercultural competency, conflict resolution, human rights, and anti-racism” [1] p. 3.

Seven years after the publication of the TRC report [1], our research team consisting of Indigenous and non-Indigenous academic scholars, Indigenous Elders, and community partners paused to reflect on what progress had been made in nursing education regarding this important Call to Action as a means of addressing racism within the Canadian healthcare system. We therefore set out to explore the literature to determine what methods and strategies have been implemented (or are currently being employed) by Canadian universities and colleges to implement the TRC Calls to Action. However, in preparing for this inquiry, we felt compelled to adopt an approach that aligned with Indigenous methodologies, understanding that Indigenous methodologies are essential to transforming and critically analyzing existing research practices with the intent to positively benefit Indigenous communities [2]. In this paper, we describe our scoping review methodology while the results of the scoping review are a focus of a separate publication. We share our journey of designing a study that embraces several core principles of Indigenous methodologies with the aim of advancing the science of scoping review methods. After introducing the co-authors and advisory team members to situate ourselves in the research, we begin with a brief review of Indigenous health research and decolonizing methodology and end with an introduction of our emergent Indigenous framework for conducting a scoping review.

### Our beginnings: forming the advisory committee (advisory circle of hearts and minds)

The primary author is an Anishinaabe scholar from Hollow Water First Nation, located in Treaty 5 territory in Manitoba. She is now residing on the outskirts of the City of Winnipeg in Treaty 1 territory. She lived and worked on the reserve as a primary care nurse in northern Manitoba for the first half of her career. Moreover, over the past 17 years, she has worked in health policy and research with the First Nations Health and Social Secretariat of Manitoba (FNHSSM), a community-based organization. She is also an adjunct professor in the College of Nursing, University of Manitoba, and was one of six in Canada who received an Indigenous Research Chair in Nursing (IRCN) award in 2020, and the first Chair to receive an award outside of an academic institution. The secondary author who was also the research coordinator is a graduate student in Kinesiology and a Red River Métis citizen who grew up in the City of Winnipeg. One of the first tasks of the IRCN was to form an advisory committee which was affectionately called the “Advisory Circle of Hearts and Minds,” paying respect to the unique contributions and perspectives that arise from both lived and learned experiences and hereafter referred to as the Advisory Circle.

The Advisory Circle consisted of Elders, Knowledge Keepers, community partners, and both Indigenous and non-Indigenous researchers. The Elders/Knowledge Keepers represented the three distinct Indigenous groups in Manitoba: First Nation, Métis, and Inuit. The primary community partner is the Manitoba Indigenous Nurses Inc., who have a vested interest in advancing Indigenous health nursing. The Knowledge Keepers and community partners generously provided their time and input since the beginning of the study.

The academic research team consisted of researchers with a wide knowledge base from Indigenous health to decolonizing education and health policy. The non-Indigenous researchers were purposely chosen by the Indigenous primary investigator for their long-standing research partnerships, respectful research practices, and exercising humility when working with Indigenous peoples and communities. Their expertise and research experience in Indigenous health is extensive and valued. The non-Indigenous members of the academic team and co-authors of this article are: [Kellie Thiessen, Josée G. Lavoie, Annette Schultz, Janice Linton, and Bekelu Negash] and from an Indigenous community health organization, [Julianne Sanguins]. The contributions of the Knowledge Keepers Geraldine Beck (Métis), Brenda Longclaws (Anishinaabe), Geraldine Shingoose (Anishinaabe) Matta Palmer (Inuk) were extremely valuable to this manuscript. Additional details of our larger Advisory Committee are provided when relevant to the discussion.

### CIHR Indigenous Research Chair in Nursing (IRCN): context

This paper and subsequent publication are situated within the program of research supported by the Indigenous Research Chair in Nursing for the Manitoba Region. This Chair is funded by the Canadian Institutes of Health Research (CIHR), the Canadian Nurses Foundation, and Research Manitoba. This special call was issued in 2019 by the Institute for Indigenous Peoples’ Health, one of the thirteen Institutes that comprise the CIHR, acknowledging the under-representation of nurse leaders in Indigenous health/research, and the significant role nurses can play in improving the health and wellbeing of Indigenous peoples. Other provincial partners have contributed to the Chairs in their respective regions. The specific objective of the Manitoba IRCN is to support the development of nursing leaders and promote cultural safety in institutional settings, including addressing institutional barriers to Indigenous health. Supporting this broader vision is the Advisory Circle of Hearts and Minds, which came together through the Manitoba IRCN and her extensive networks.

### Historical context surrounding Indigenous peoples and research

In 1997, the Royal Commission on Aboriginal Peoples (RCAP) reported that the gathering of information on Aboriginal people and its subsequent use was inherently political [3]. They also noted that Indigenous peoples historically have not been consulted about what information should be collected, who should gather that information, who should maintain it, and who should have access to it [3]. As such, the information gathered may or may not have been relevant to the questions, priorities, and concerns of Aboriginal peoples [3], p. 498). This report called for fundamental changes to how research is conducted with Indigenous people in Canada. Following this landmark report, the principles of OCAP® emerged [4]. Originally coined by the Steering Committee of the First Nations-led Regional Health Survey, the OCAP® principles have become a mechanism to assert First Nations self-determination in research and the acronym is now a registered trademark by the First Nations Information Governance Centre in Ottawa [5]. Key points related to these principles are the collective ownership of data and information; First Nations control over research and information; First Nations’ management of access to their data and physical possession of the data.

Further, in 2013, the United Nations Declaration on the Rights of Indigenous Peoples (UNDRIP) affirmed the rights of Indigenous Peoples to enjoy and practice their cultures and strengthen their social and political institutions, and in 2015, the Truth and Reconciliation (TRC) of Canada called for increased control of Indigenous Peoples over their institutions, including research related to them [6]. These landmark documents echoed the longstanding plea by Indigenous scholars and communities for greater involvement in research and greater inclusion of Indigenous worldviews and perspectives in research [7, 8]. We believe that any written account or research related to Indigenous peoples should not be exempt, whether it involves the primary or secondary collection of narratives, quantitative data, or the synthesis of published literature, such as scoping or systematic reviews. It was therefore imperative we adapt the mainstream scoping review methodology so that it is relational, aligned with Indigenous knowledge and research methodologies, and led by Indigenous people.

### Grounding our work

While Western methods of conducting literature reviews have historically been a sole academic endeavor, the research team fully acknowledged the value of a relational approach or the use of community-based participatory (CBPR) methods in previous endeavors to decolonize the systematic literature review process [8, 9]. However, in these instances, we noted that advisory or expert groups were not engaged throughout the entire review process, or their level of involvement was not well documented. Having no established template or guideline to follow for a scoping review incorporating an overarching Indigenous framework, our research team felt it was important to make space for Indigenous leadership, detail the process, and create opportunities for Indigenous peoples to have meaningful involvement in research that reflected their diverse values, identity, lived experiences, stories, and traditions [10].

The Advisory Circle began meeting in 2020, and after some discussion, a decision was made to start with a review of current practices in nursing education as it pertained to Call to Action #24 [1]. The Advisory Circle felt this was an essential first step and a logical place to start. We acknowledged the importance of conducting a reflective review to assess the existing evidence, aiming to comprehend past initiatives and identify the necessary steps to further progress in fostering reconciliation within nursing education. The academic team acknowledged the importance of adapting common approaches to reviewing the literature, with the specific intention of engaging the Advisory Circle early and meaningfully to begin our work “in a good way”. This term was used by the Knowledge Keepers to describe the ceremonial and relational aspects of research, but it also refers to researchers' ethical responsibility to ensure that Indigenous knowledge and people are not exploited and that the methods and methodology are consistent with respectful research practices [11].

### Reframing and incorporating Indigenous methodology

Multiple Indigenous approaches guided our research process: Kovach’s Nêhiyaw conceptual framework [7], Smith’s decolonizing methodology [8, 12], and Phillips-Beck Anishinaabe framework [10]. Although the primary investigator and first author’s background is Anishinaabe, Kovach’s Nêhiyaw (Cree) conceptual framework was remarkably relevant and a useful starting point [7]. Kovach’s conceptual framework was grounded in Nêhiyaw epistemology and acknowledged the holistic, non-linear, and relational aspects of being. It included six phases where we envisioned an Indigenous lens could be positioned: (1) researcher preparation, (2) research preparation, (3) decolonizing ethics, (4) gathering knowledge, (5) making meaning, and (6) giving back [7]. While we do not go into any detail explaining what each of these phases are, we embrace the circular nature of this model which allows for fluid movement within and across these domains. In addition to finding those points of intersection to position an Indigenous lens, we also reframed Arksey and O’Malley’s methodological framework [10] to align with our processes which were circular and reciprocal, as opposed to the generally accepted linear representation of the Western research process.

Key elements of Smith’s [8] Indigenous methodology that we took note of included: challenging traditional power structures; inclusion of Indigenous peoples, voice, and worldview; grounding the work in ceremony and/or incorporation of cultural protocols; ensuring the research was of benefit to Indigenous peoples; and the importance of contextualization, particularly as it pertains to colonization and the history of Indigenous Peoples. Smith [8] emphasized that history and context are important in research as colonization remains very much a contemporary issue, and the effects of intergenerational trauma and racism still reverberate today. Further to that, Kovach [7] advised that we needed to move beyond simply critiquing colonization to challenging the colonial policies and processes that exist in academic institutions today. From our perspective, this manifested in our research methodology through a governance structure, introduced earlier as the Advisory Circle that aimed to elevate Indigenous Knowledge Keepers and their voices in research that could potentially impact nursing education in the future.

### Anishinaabe framework guidance

**Fig. 1:**
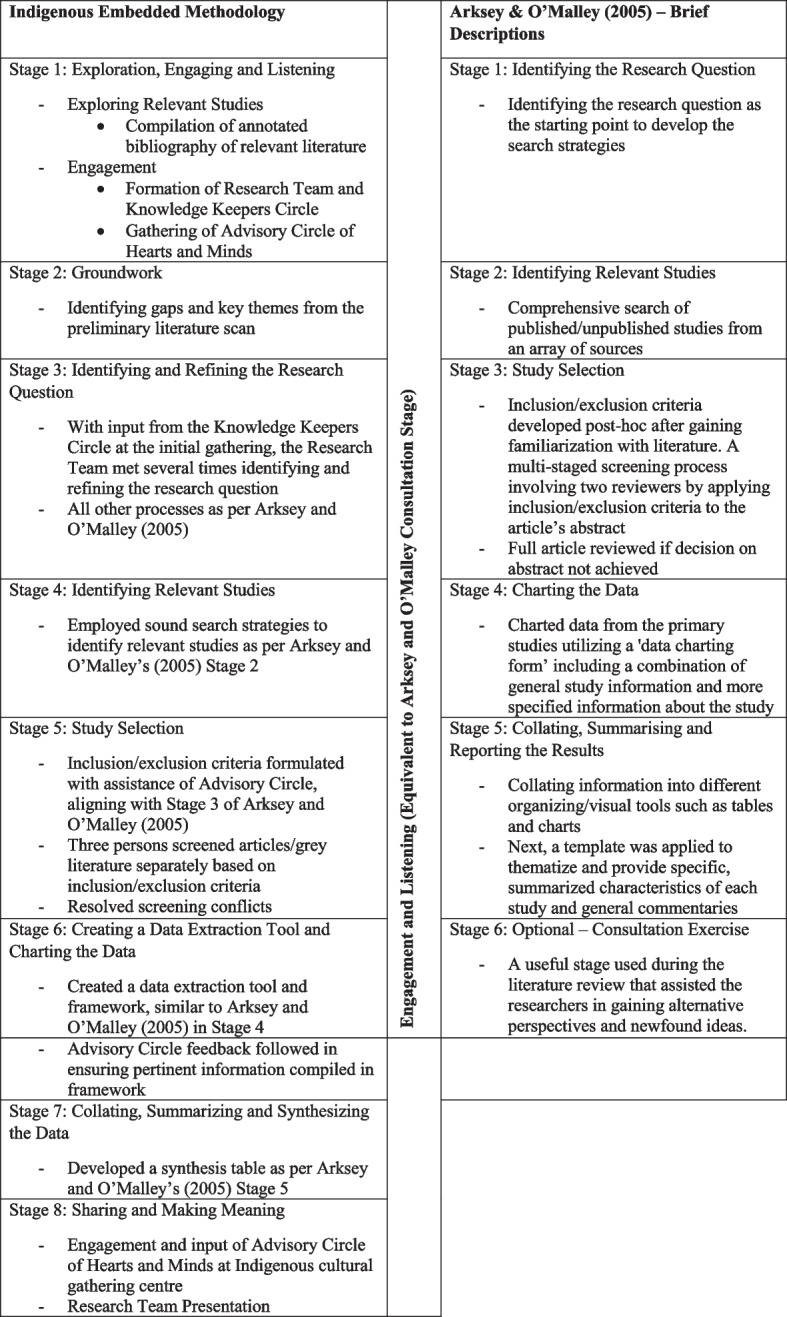
Mapping our Indigenous Pathway on Arksey & O’Malley’s (2005) 5 Stages

Lastly, we utilized Phillips-Beck’s [13] Anishinaabe framework from her doctoral dissertation to frame the study. The Anishinaabe Framework includes two tenets that are founded in two powerful Indigenous teachings: *Bi-zin-doi-zhen*, (listen intently) and *Wewini Anokiiwin* (work properly, in a good way) which describes her vision of the space where Western quantitative research and Indigenous methodology intersect, and the space where decolonization occurs (11). The first tenet, *Bi-zin-doi-zhen*, involves listening to Indigenous people’s perspectives on the research topic, followed by the second tenet *Weweni Anokiin*, to do the work properly which can involve both Indigenous and Western methods, and circles back again to *Bi-zin-doi-zhen*, to listen intently once again to Indigenous voices in the interpretation of findings and “within” by reflecting on the research journey. This circular process is mapped on the traditional Western process which is generally linear in its general application (Fig. 1).

In the Anishinaabe framework, *Bi-zin-doi-zhen* is actioned by building relationships with Knowledge Keepers, Elders, and Indigenous communities and beginning the work in ceremony (to welcome ancestors to our work) and including their perspectives in framing research questions and at key points in the research [10]. We felt that Knowledge Keepers and Elders’ perspectives in the preliminary stages of research were important, as their experience and understanding of Indigenous health and history best position them to contextualize the issues that impact their people [13]. It was also important to capture their expertise in the scoping review design, particularly in the preliminary stages of the research, as it allowed the researchers an opportunity to prepare for the research, to listen, learn, and to take direction such as when ceremony and protocols were appropriate. The second tenet of the Anishinaabe Framework, *Weweni Anokiin*, involves doing research in a proper way using solid robust research approaches, regardless of whether they are considered Indigenous or Western methods [10]. In our case, we followed generally accepted scoping review practices. While Western methods may be used to collect or analyze data, we made a concerted effort to involve Indigenous people and perspectives at every stage of the research process through meaningfully engaged dialogue [13].

The last tenet “*Bi-zin-doi-zhen*” resurfaces at the end of the research, affording the research team another opportunity to gather in the ceremony, and receive feedback or advice from Indigenous voices to guide the final phases of the research. At this point, we involved the Knowledge Keepers and Elders in the interpretation of the study findings. We listened to learn how these findings are important and relevant to Indigenous Peoples and how we might share these findings with various communities of interest.

### Mapping the Indigenous scoping review methodology to Arksey and O’Malley 5-Stages

Arksey and O’Malley’s [10] approach to the scoping review method has seen a variety of adaptations aimed at refining and documenting the scoping review method [14, 15]. Interestingly, the original five-stage approach (and optional sixth stage) to scoping reviews remained rather constant over the years, which included: (a) identifying the research question; (b) identifying relevant studies; (c) study selection; (d) charting the data; (e) collating, summarizing and reporting the results; and (f) an optional consultation stage [10]. In our Indigenous-informed adaptation, we retain these five stages, but after some reflection and discussion among the research team, we envisioned three additional stages to allow for the preparatory work. This preparatory work was important to ensure that Indigenous voices informed the scoping review, and to find those places where we could incorporate elements of Indigenous methodologies.

**Fig. 2:**
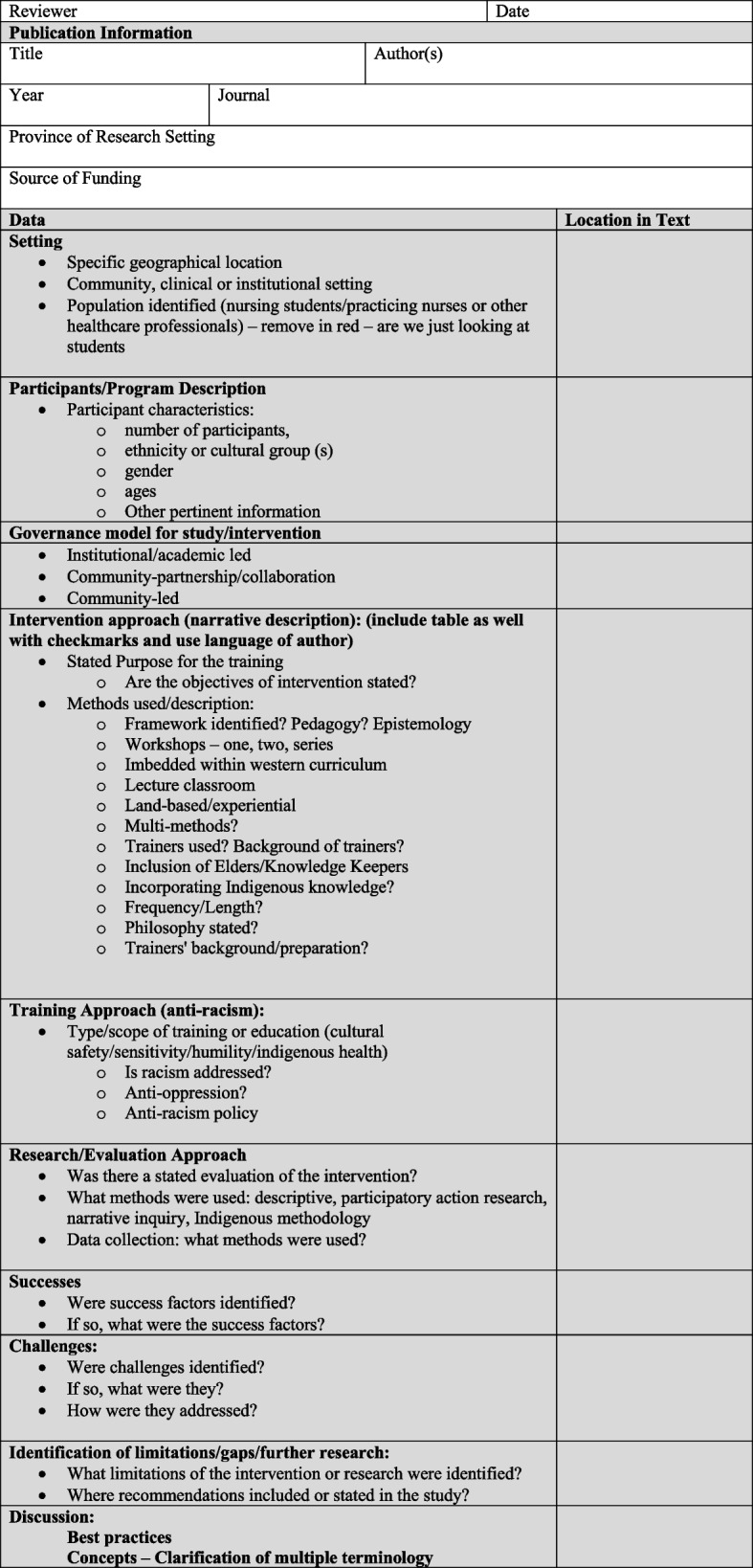
Indigenous Scoping/Narrative review: draft environmental scan/assessment framework

The three additional phases of this Indigenous-embedded methodology involving the preparatory work are in order: Exploration/Listening (stage 1); Groundwork (stage 2); and Sharing and Making Meaning (stage 8). Stages 3 to 7 of our Indigenous embedded methodology correspond directly to stages 1 to 5 of Arksey and O’Malley’s scoping review methodological framework [9]. Engagement, or consultation as the optional stage in Arksey and O’Malley’s framework, is central to our approach. However, we purposely refrained from using the term “consultation” as this term has been criticized by Indigenous peoples for not accurately capturing and acknowledging their meaningful and active participation [12]. We used the terminology of “engaging and listening” [12] to appropriately reflect the Anishinaabe Framework. As illustrated in Fig. 2, our Indigenous-informed scoping review process utilizes eight stages, mapped alongside Arksey and O’Malley’s five stages [10]. In keeping with an Indigenous methodology, our scoping review approach is circular in nature but depicted in table format. While Arksey and O’Malley [10] encourage an iterative approach to scoping reviews, their framework did not allow for an iterative approach that privileged Indigenous voices and/or other voices to circle back in at key points. We will describe the 8 stages in the following section.

## Scoping review description: an eight-stage Indigenous-informed scoping review methodology

### Stage 1: exploration, engaging, and listening

#### Exploring relevant studies

Preliminary and exploratory searches of academic peer-reviewed literature were conducted by the research coordinator in 2020 with a focus on nursing education and Indigenous people, or programs supporting Indigenous nursing students. This early-stage search was supported by the Indigenous Health Liaison Librarian at the University of Manitoba who had substantial experience working with Indigenous literature. From this search, the research coordinator compiled an annotated bibliography of studies or articles from Canada, the USA, Australia, and New Zealand as countries with similar histories of colonization. The annotated bibliography informed our early research team discussions, including how we might take what we learned to our Knowledge Keepers Advisory Circle. In this stage, in contrast to Arksey and O’Malley’s [10] first stage of identifying the research question, we explored several possible questions that could be the focus of our scoping review.

#### Engagement: the first gathering of the advisory circle of hearts and minds

Introduced earlier, the Advisory Circle of Hearts and Minds consisted of Elders/Knowledge Keepers with a wide array of perspectives (Anishinaabe, Cree, Métis, Inuit), academic researchers, and community partners that came together periodically depending on availability or the purpose for the meeting. As of June 2023, there were 13 members in total, but the number of members attending meetings fluctuated anywhere from 8 to 12. In September 2020, the Advisory Circle came together as an entire group virtually, due to the reimplementation of COVID-19 restrictions in Manitoba that interrupted initial plans to host an in-person meeting at a cultural center located out on the land. Meeting virtually posed some challenges in establishing those personal connections and incorporating ceremony and protocols to start us off in a good way, but it was imperative that the research began in the ceremony. A prayer and smudge were offered by one of the Knowledge Keepers from her home location, and after presenting via a virtual platform what we found in the preliminary literature scan, we proceeded with a loosely facilitated discussion. We provided time and space for the Knowledge Keepers/Elders to provide input on possible foci and guiding questions for a deeper dive into the literature.

Guiding questions planned for reflection included: (1) what information is necessary for all nursing students to know about Indigenous health/people? (2) What role do you see Knowledge Keepers playing in nursing education? What knowledge can be shared? (3) How can we share this knowledge in a good way, which honors Indigenous ways of knowing, values, and ethics? However, the conversation gravitated toward a discussion on colonization, particularly how it has impacted the health and well-being of Indigenous people in Canada. The Knowledge Keepers also addressed the complete neglect by Canadian educational systems in teaching an accurate history of colonization to all citizens. They emphasized the importance of initiating this education at the secondary school level and extending it to all newcomers to Canada, not solely limited to nursing students. Despite the Knowledge Keepers not being aware of Smith’s [16] position that research includes a critique of colonization or having any prior knowledge of her work or other scholars in this regard, the conversation went naturally in this direction. The insights they shared were based solely on their own observations and experiences.

This gathering was vital to our process to determine the Knowledge Keepers and Elders perspectives on the research topic and most likely the first opportunity for Knowledge Keepers and Elders to share their personal experiences in the healthcare system and about encountering racism. We simply provided a safe space for the Knowledge Keepers and Elders to articulate their perspectives freely, but we eventually circled around to a conversation about the future of nursing education. Collectively, the research team learned about their interactions with the nursing profession directly and heard about opportunities for improvement, starting with how we currently educate our nursing workforce. They emphasized that the present nursing educational system is situated in a very colonial institution, dominated by White and Western perspectives [11], with no opportunities for their voices to be heard. We held on to this knowledge, as it would inform our discussions in the later stages. An important outcome of this meeting was greater clarity regarding the primary focus of the scoping review.

### Stage 2: groundwork

The next stage consisted of regular meetings with the Advisory Circle, occurring bi-weekly through virtual platforms, due to the COVID-19 pandemic public health restrictions. The preliminary search and summary of the literature conducted by the research coordinator were compared to the themes voiced by the Knowledge Keepers at the initial gathering. Gaps in the literature, how to uncover unindexed sources, and recommendations for a focused search were discussed by the research team during these meetings. These discussions and regular engagement sessions were important for capturing both Indigenous and academic perspectives as well as considerations on how this research may impact Indigenous Peoples. While there is alignment with Arksey and O’Malley’s [10] consultation phase, unique to our second stage is the ongoing and iterative process of listening to and including diverse voices in the beginning, and at varied points in the research process. By drawing on the guidance of the Advisory Circle of Hearts and Minds, we remained consistent with Indigenous methodologies proposed by Kovach [7] and the Anishinaabe framework (10) to include and value evidence from diverse sources.

### Stage 3: identifying and refining the research question

Based on the preparatory work during stages 1 and 2, four priority areas were identified by the research team as a focus for the scoping review: (1) a critical appraisal of terminology, use and application surrounding the concepts of cultural competency and safety; (2) an assessment on what is currently happening in nursing education programs in Indigenous health education**,** including the core components/content for this education; (3) anti-racism policies and initiatives in nursing education and lastly, (4) how Indigenous Peoples are positioned within these frameworks. These four priority areas stemmed from the conversations during our initial Advisory Circle gathering, and after further discussion with the research team, we agreed on the following research question to guide our scoping review:

#### What methods and strategies are employed within Canadian university/college entry-level nursing education programs specific to delivering health care to Indigenous peoples in Canada?

The dialogue ends in solidifying the research question in this stage, aligning with *Weweni Anokiin*, the second tenet of the Anishinaabe framework [13]. Moreover, it is consistent with the description by Berryman et al. [17] of a culturally responsive, humble approach. The non-Indigenous academic researchers continuously provided valued input during the research process, however, their understanding and appreciation for Indigenous-governed research was observed in their willingness to listen and position themselves as outsiders and co-learners, whether that be in meetings, or in the development of tools and parameters for the scoping review. As well, this approach acknowledged the importance of conducting research that has tangible implications for Indigenous Peoples starting at the preliminary research priority-setting phase and in developing research questions [8, 16]. Overall, the formation of the research question was informed by the *engaging and listening* stage, demonstrating its practical use throughout the entire research process. While this stage is similar to Arksey and O’Malley’s [10] first stage, engagement and listening are emphasized in our methodology. Hearing the diverse Indigenous perspectives helped craft the research question and establish additional priority areas for the IRCN; all of which remained consistent with the second tenet of the Anishinaabe framework, *Weweni Anokiin* [10].

### Stage 4: identifying relevant studies

After finalizing our research question, we circled back to Arksey and O’Malley’s [10] methodology to identify relevant studies. This is where our fourth stage corresponded with Arksey and O’Malley’s second stage to identify relevant studies. In this stage, with the help of the librarian, we conducted a comprehensive review of the literature. We searched through electronic databases for published academic and grey literature, hand-searched the table of contents of non-indexed sources, and conducted Google searches for possible reports not found in the electronic databases. This also corresponded with Phillips-Beck’s [13] second tenet of the Anishinaabe Framework “Weweni Anokiin”, *to do the work properly* using methods that are consistent with Western robust research practices. This stage allowed for Western methods to be used to search for data with the full confidence that Indigenous voices and perspectives had been central to the formation of the research question.

#### Search strategy

Our search strategy remained consistent with Arksey and O’Malley’s [10] methods for conducting a scoping review, apart from test searches that were carried out by the librarian on the topic of nursing education and Indigenous people. These initial searches included grey literature and manual searches through non-indexed journals to ensure all possible sources relevant to our topic were captured. As such, several pages of links to a wide range of documents on nursing education, nursing associations, and policy statements were included for further review.

### Stage 5: study selection

Through the course of our bi-weekly meetings, the Advisory Circle was also involved in helping formulate the inclusion/exclusion criteria for the initial screening of the literature. Six inclusion/exclusion parameters were agreed upon and described in more detail in the scoping review publication.

#### Screening

Review of titles and abstracts and screening were done following standard scoping review methodology, and consistent with good research practices for conducting a scoping review as per Arksey and O'Malley [10]. The 3 members of the research team individually conducted these. However, one key difference in this stage was the necessity of conducting a full text/document screening (or second-level screening) due to vaguely written abstracts, or the absence of an abstract due to the document being a report or a chapter found within a larger document. At this stage of review, the reviewers also extracted relevant quotes and compiled a file of excluded articles that were deemed of interest or relevance to our study that could be used for the writing of the manuscript.

### Stage 6: creating a data extraction tool and charting the data

With final inclusion decisions made, in collaboration with the Advisory Circle, we developed a data extraction framework [see Additional file 1] to organize relevant information from each article/document. We applied the data extraction framework when analyzing the articles and the grey literature. We had three independent screeners, who came together and made decisions to include/exclude when there was disagreement. One key difference from standard scoping review practice was to provide an Indigenous lens to the data extraction tool to ensure that the most pertinent information would be captured from eligible articles.

### Stage 7: collating, summarizing, and synthesizing the data

This synthesis and analysis of the data involved a combination of both Indigenous and Western-based approaches to align with Arksey and O’Malley’s [10] stage five to collate, summarize, and report the results. The previously mentioned, the data extraction tool was collated into a synthesis document and followed similar Western methods as per Arksey and O’Malley’s [10] framework. However, in addition to summarizing themes derived from the literature, our synthesis included general commentaries, observations, and perspectives obtained from the Advisory Circle, as opposed to creating a general summary based solely on the 3 reviews’ perspectives. This process allowed for Indigenous voices to be included in reporting our results and within the discussion sections of our scoping review manuscript.

The synthesis table organized the data qualitatively through summarized descriptions of each article, including our own observations, and quantitatively with a checklist identifying if the articles and grey literature documents included relevant secondary data, such as whether the goal of the interventions had been specified as cultural safety, anti-racism, or otherwise. Despite both the synthesis table and data extraction framework being Western methods of analyzing the research results, the development of these frameworks, in collaboration with the Advisory Circle continued to engage Indigenous peoples. Combining both Indigenous perspectives and Western methods such as the Preferred Reporting Items for Systematic Reviews and Meta-Analyses extension for Scoping Reviews Checklist (PRISMA-ScR) [18] guidelines in the data synthesis and analysis phases are important elements of our Indigenous-informed methodology for conducting a scoping review.

### Stage 8: sharing and making meaning

#### Cedar Lake Ranch workshop presentation

The Anishinaabe framework came full circle in the culmination of the data collection process, as both the primary investigator and research coordinator presented the preliminary research findings to the Advisory Circle at a second gathering held on the land at Cedar Lake Ranch (an Indigenous cultural gathering space in Manitoba, Canada). In addition to the Knowledge Keepers and research team, others invited to join the meeting included an Indigenous community health nurse, a retired Indigenous nurse, and an Indigenous PhD student. The gathering opened with a prayer and ceremony, as per our traditional protocols, followed by an acknowledgment of each member of the Advisory Circle. The research findings were later presented by the research coordinator which included the scoping review parameters, and a brief overview of our methods and results flow diagram.

The second part of the gathering included engaging the attendees in reviewing the preliminary findings. We created flip chart stations in different places in the large circle room where we had written down brief summaries of each of the themes we had identified in the literature. We provided the attendees an opportunity to provide both written and oral feedback, provide relevant context, or voice their opinions on the findings. The primary investigator and research coordinator listened to the attendees as they reflected upon the methods/strategies that were employed by Canadian institutions to educate nursing students about Indigenous Peoples. They offered further insights that could inform the findings of the scoping review. This activity highlighted the circular nature of the Anishinaabe framework [10] by circling back and “listening” again to the Indigenous Knowledge Keepers/Elders and attendees’ voices once again. In this final stage, we not only considered the findings of our review, but we also reflected on the research journey and why this research was important to Indigenous Peoples.

#### Research team presentation

The research team members who were unable to attend the Cedar Lake Ranch presentation gathering were invited to attend a similar, condensed version of the workshop virtually on July 28, 2021. The research team members similarly offered their thoughts verbally regarding the methods/strategies employed in the literature, as well as advice for the scoping review written component and discussion authorship. This presentation, in conjunction with the Cedar Lake Ranch gathering, was an extremely valuable opportunity to listen intently to both the Knowledge Keepers and academic team members.

## Discussion

Many of our discussion points have been articulated throughout the documentation of this scoping review process. We utilized Indigenous research practices that included positioning an Indigenous lens at key points in the research process and purposefully reclaimed Indigenous space in research by creating a governance structure that included Indigenous people to guide our work. This was to ensure that the 8-stage Framework for conducting scoping reviews remained consistent with Indigenous methodologies [7, 8]. Our Indigenous-embedded methodology stressed the importance of taking a relational stance and engaging Indigenous people early. This approach is consistent with Indigenous scholars such as Michal Hart [19] who stressed how important it was to incorporate the perspectives of local community values and aspirations and to approach research in a manner that is consistent with Indigenous worldviews. Our Indigenous embedded methodology, set out to prepare for the research by listening first, as per Phillips-Beck’s [13] *Bi-zin-doi-zhen,* to “listen intently”, the first tenet of the Anishinaabe framework and Kovach’s [7] first phase of her Nêhiyaw framework. It also heeded Smith’s [20] advice to contest oppressive power differentials that historically exist within research and within institutions. For our scoping review study, this translated to a governance structure that included the perspectives of Knowledge Keepers. The guidance provided by the Knowledge Keepers included consideration of the potential research questions, search strategies, and findings based on their knowledge of existing nursing educational practices and experiences in the healthcare system.

Building on Arksey and O’Malley’s methodology [9], our Indigenous-informed scoping review methodology incorporated a preparatory phase that began in the ceremony, as per local customs and protocols [21], followed by listening to and engaging with Indigenous voices to hear their perspectives on the research topic. The preparation stage allowed time to dialogue with Indigenous collaborators which is also consistent with Indigenous methodologies to center the voices and meaningfully engage with Indigenous people [7, 8, 19, 22]. This contrasts with Arksey and O’Malley’s [10] scoping review method that identifies the research question as the initial stage.

Our Indigenous-embedded methodology is also consistent with Tynan and Bishop’s [23] relational literature review process. They contend that the literature review does not necessarily start with literature, but begins with your own relationships with people, places, and knowledge. We concur. A relational literature review process shifts the purpose of a literature review (the scoping review included) to not only extract or find gaps in the literature, but to benefit our relations as well. This relational approach obligates the research and researchers to be accountable to our relations or to follow up on what the implications are, as advocated by prominent Indigenous scholars such as Michael Hart [19], Linda Smith [8], and Shawn Wilson [24]. Not only did we have the willingness to build and form those relationships with Indigenous people, but with their permission, we were able to include their perspectives in sharing the findings more broadly in other local policy tables and in research forums. In this case, we were able to elevate their voices to potentially influence decisions at local institutions and spaces where the primary investigator has a voice. In this way, we were able to demonstrate accountability to our relations to follow through with what we learned. Our Indigenous-embedded methodology review also demonstrated how this may be accomplished in what is generally considered a relatively benign undertaking such as a scoping review.

Giving back and sharing findings in a manner that is understandable to Indigenous Peoples and use of cultural practices and protocols was also evident at all gatherings of the Advisory Circle particularly those that took place on the land of the Cedar Lake Ranch. These practices aligned with Kovach’s [7] Indigenous methodology to give back in a meaningful and respectful way and consistent with the final quadrant and second Tenet “Weweni Anokiin” of Phillips-Beck’s framework [13]. Elevating the voices of Indigenous Knowledge Keepers and incorporating local protocols and practices can seem to be a natural practice for the Indigenous scholars but may take a concerted effort by others who desire to do work in “a good way”. The Knowledge Keepers/Elders and attendees’ voices provided valuable feedback on the entirety of the research process and offered their perspectives and input in conducting the final research steps. Overall, engagement was an integral part of the entire research process, particularly in the Cedar Lake Ranch gathering but at all critical points in the research that allowed for a more intimate and nuanced discussion with Indigenous peoples.

Although Phillips-Beck’s Anishinaabe [13] Framework was originally designed to guide quantitative researchers to find those areas of intersection where they could build in elements of Indigenous methodology, the framework itself can be useful in conducting literature reviews that utilize the typical research process for any purpose. As you can see in Fig. 1, each phase of the Western research process (Research Planning/Data Collection/Data Analysis/Knowledge Translation) is superimposed upon the two circular Tenets of the Anishinaabe Framework: (1) Listen intently and (2) work in a good way. Each quadrant of the Anishinaabe Framework corresponds to a phase in the Western research cycle and includes intersecting Action Pillars where elements of Indigenous methodology can be incorporated. For example, Action Pillar 1, attached to Tenet 1, reminds us of the importance of building relationships, meaningful engagement, and grounding the research in Indigenous knowledge by incorporating Indigenous perspectives to understand the topic. As the research progresses to the data analysis/interpretation phase, we are again reminded to listen intently once again to understand the data from an Indigenous lens. Action Pillar 3 in this phase encourages us to include Indigenous knowledge, perspectives, and worldviews once again to contextualize the findings, so that it reflects the realities of Indigenous people. Context is important [8] and situating the research within the context of the data source as we do in quantitative research (literature reviews included) is a crucial consideration for researchers engaged in Indigenous research methods and frameworks [25].

### Challenges/operational issues

Challenges and operational issues that occurred were due predominantly to the COVID-19 pandemic. For instance, the initial September gathering was intended to be in-person at an Indigenous cultural gathering site. However, due to COVID-19 restrictions, the gathering was instead held over Zoom, a virtual platform. This somewhat restricted our ability to engage in cultural ceremony and spiritual prayer in a way that honored Indigenous worldviews, ethics, and values. The virtual platform made it difficult for Knowledge Keepers/Elders to be engaged at every meeting. However, when restrictions allowed for in-person gatherings, they were brought back in at key points in the research.

### Successes

It is worth reiterating that one of the notable successes in this research process lies in the composition of the research team. The Advisory Circle of Hearts and Minds is comprised of diverse perspectives and backgrounds in both academia and the community, both Indigenous and non-Indigenous, and sometimes included members who joined temporarily or were free to provide their input and circle in and out of the conversations as they felt inclined. Additionally, the research was led by an Indigenous principal investigator who is employed by an Indigenous, community health and advocacy organization (FNHSSM), further providing an additional layer of insight to one of the most pressing research topics involving nursing and Indigenous Peoples. This combination of rich and nuanced outlooks on research has been integral in every sequential or circular step of the research process. Another important success was following the advice of the Advisory Circle in keeping the research focused and reframing the scope of the research question.

## Conclusion

This paper highlighted how Indigenous methodologies can inform the scoping review process. We expanded Arksey and O’Malley’s [10] 5-stage methodological framework to an 8-stage framework by incorporating several elements of Indigenous methodologies. Some of these key elements included building relationships and engaging Indigenous peoples early, forming a governance structure in the form of an Advisory Circle to guide the research, incorporating cultural protocols and practices into the research process, and active involvement of Indigenous perspectives in critically reviewing and interpreting the findings. Although this example is clearly a demonstration of Indigenous-led research, it does not preclude Western-trained researchers from applying this methodology to any scoping review. Our Indigenous-informed methodology utilized all stages of Arksey and O’Malley’s [9] framework to ensure that robust research practices are followed but included one key fundamental difference: the optional “consultation”, stage in Arksey and O’Malley’s [10] is a central component of the 8-stage model and is interwoven at key points of the research process. By conducting our scoping review in such a way, we were able to capture the perspectives of Indigenous Peoples from the beginning in forming the research question and capturing their insights about what is important to learn from the literature to circling back near the end to hear once again Indigenous voices in reviewing our findings. It is our hope that by sharing our process of conducting a scoping review rooted in Indigenous-centred practices, we create space for Indigenous perspectives to identify themes, and gaps or map the literature on evolving and emerging topics that are relevant to Indigenous peoples. Through sharing both our journey of designing an Indigenous scoping review approach, along with describing our scoping review methodology, we aimed to provide an additional pathway that others can consider if they wish to do research in a good way.

### Supplementary Information

Supplementary Material 1: Figure S1. Anishinaabe Framework-The intersection of decolonizing research and Western research cycle.

## Data Availability

Not applicable.

## Abbreviations

OCAP: Ownership, Control, Access and Possession
RCAP: Royal Commission on Aboriginal Peoples
UNDRIP: United Nations Declaration on the Rights of Indigenous Peoples
TRC: Truth and Reconciliation Commission
IRCN: Indigenous Research Chair in Nursing
PRISMA-ScR: Preferred Reporting Items for Systematic Reviews and Meta-Analyses extension for Scoping Reviews Checklist
FNHSSM: First Nations Health and Social Secretariat of Manitoba
UM: University of Manitoba
COVID-19: Coronavirus disease of 2019
