# Supplementary material for: An Indigenous-informed scoping review study methodology: advancing the science of scoping reviews
Source: Syst Rev. 2024 Jul 15;13:181. doi: 10.1186/s13643-024-02586-1 (PMC11247733; doi:10.1186/s13643-024-02586-1)
Supplement: Supplementary file 1 — Supplementary Material 1: Figure S1. Anishinaabe Framework-The intersection of decolonizing research and Western research cycle. [file 13643_2024_2586_MOESM1_ESM.docx]

**Additional File 1**

**Supplementary information.**

**Figure 1: Anishinaabe Framework - The intersection of decolonizing research and western research cycle**

**Tenet 1**

**Tenet 2**

ACTION PILLAR 4

ACTION PILLAR 3

ACTION PILLAR 2

ACTION PILLARS 1

Data Analysis/Interpretation

Research Planning

Knowledge Translation/Dissemination

Research Coordination/Data Collection

*Adapted from: Phillips-Beck, W. (2021). A Decolonizing Approach in Population Health Research: Examining the Impacts of the Federal Out for Confinement Policy on Maternal and Child outcomes in First Nation Communities in Manitoba. University of Manitoba. Winnipeg.
